# Death

**Published:** 1879-10

**Authors:** 


Persons may have been put in a coffin before they were perfectly dead, but it is absurd to suppose that life is possible after an interval of perfect seclusion from fresh air from the time of fastening the lid until the coffin reaches its last resting-place. The action of the gases in the cadaver will naturally and sufficiently explain all the appearances observed on occasions of opening the coffin after burial. The description which Hippocrates, the "Father of Medicine," gave of death over two thousand years ago, has never been improved upon. "The forehead wrinkled and dry; the eye sunken; the nose pointed, and bordered with a violet or black circle; the temples sunken, hollow, and retired; the lips hanging down; the cheeks sunken; the chin wrinkled and hard; the color of the skin leaden or violet; the hairs of the nose and eyelashes sprinkled with a yellowish white dust." This is as to the face; and when all observed, we may know that that face can never be lighted up to life again. But there are other proofs which do not leave the shadow of a doubt, as when the heart ceases to beat; the skin is pale and cold; a film is over the eye; the joints, first rigid, have become flexible; and a dark greenish color begins to form about the skin of the abdomen, the infallible sign of beginning corruption. But as we would have it done to us as the last request, let us with the utmost willingness allow the poor helpless, unresisting frame remain at least forty-eight hours under the unfastened lid after the surest proof of all has been noticed, the cessation of all movement of the chest and abdomen, for then the breath of life has gone out forever. The moments immediately preceding death from disease are probably those of utter insensibility to all pain, or of a delightful passivity, from that universal relaxation of every thing which pertains to the physical condition. Hence Louis XIV. is reported to have died saying: "I thought dying had been more difficult." The greatest surgeon of all ages, William Hunter, while dying said: "If this be dying, it is a pleasant thing to die." Dear reader, may you and I so live, that in the practice of bodily temperances and moral purities, death may be to us the gate of endless joy and sinless bliss.

